# Effect of CNT incorporation on PAN/PPy nanofibers synthesized by electrospinning method

**DOI:** 10.3906/kim-1911-49

**Published:** 2020-08-18

**Authors:** Atike İNCE YARDIMCI, Metin TANOĞLU, Selahattin YILMAZ, Yusuf SELAMET

**Affiliations:** 1 Department of Material Science and Engineering, İzmir Institute of Technology, İzmir Turkey; 2 Technology Transfer Office, Uşak University, Uşak Turkey; 3 Department of Mechanical Engineering, İzmir Institute of Technology, İzmir Turkey; 4 Department of Chemical Engineering, İzmir Institute of Technology, İzmir Turkey; 5 Department of Physics, İzmir Institute of Technology, İzmir Turkey

**Keywords:** Carbon nanotube, electrospinning, polyacrylonitrile/polypyrrole nanofibers

## Abstract

In this study, carbon nanotubes (CNTs) added polyacrylonitrile/polypyrrole (PAN/PPy) electrospun nanofibers were produced. Average diameters of the nanofibers were measured as 268 and 153 nm for 10 and 25 wt% of PPy contents, respectively. A relatively higher strain to failure values (23.3%) were observed for the low PPy content. When as-grown CNTs (1 and 4 wt%) were added into the PAN/PPy blends, disordered nanofibers were observed to form within the microstructure. To improve the interfacial properties of CNTs/PAN/PPy composites, CNTs were functionalized with H_2_SO_4_/HNO_3_/HCl solution. The functionalized CNTs were well dispersed within the nanofibers and aligned along the direction of nanofibers. Therefore, beads formation on nanofibers decreased. The impedance of the nanofibers was found to decrease with the PPy content and CNT addition. These nanofibers had a great potential to be used as an electrochemical actuator or a tissue engineering scaffold.

## 1. Introduction

There is a great research and industrial interest to use carbon nanotubes (CNTs) as nanofillers to obtain polymer composites with improved mechanical, electrical and thermal properties [1–5]. Because of their superior mechanical properties such as modulus, strength and thermal and electrical conductivity with their low weight, they have been attractive candidate for composite systems [6,7]. The main problem for using CNTs as filler materials is their poor dispersion in matrix. Dispersion and alignment of CNTs influences directly the mechanical properties of final composite. Agglomerated CNTs cause defect sites, porous regions, and crack initiation points, etc. which deteriorate the mechanical properties of composite. There are several techniques to achieve a good dispersion of CNTs in a polymer matrix such as, high speed mixing [8], in situ polymerization [9], chemical functionalization [10–12] and high shear mixing [13]. Surface functionalization of CNTs has been found be an effective method to obtain a good CNT dispersion and enhance the bonding between the CNTs and polymer matrix.

In addition to their excellent electrical and mechanical properties [14], CNTs show electromechanical actuation behavior. This electromechanical behavior makes CNTs a good candidate for application such as sensors and actuators [15]. By incorporating CNTs, actuation response of the polymer is significantly improved and actuation occurs at lower voltages as compared to those of without CNTs [16]. It was also revealed that CNTs added into conducting polymers may increase actuation strain and conductivity, therefore, electrochemical efficiency in the actuation is provided [17]. Courty et al. [18] reported substantial dielectric anisotropy by aligning CNTs in the polymer and the actuation response was due to the large anisotropy in nanotube polarizability. Zheng et al. [19] fabricated polypyrrole (PPy)/CNT laminates by electrochemically polymerizing PPy on a glassy carbon substrate. Incorporation of the CNT sheets resulted in an increase of strength for both transverse and longitudinal directions and enabled actuation at much higher loads, as compared to those for electrolyte-contacting neat PPy. Electrical conductivity was also improved with the CNTs content.

Park et al. [20] added flouro group bonded functional CNTs slurry to the PAN solution. An enhancement in mechanical properties of the carbon fibers was observed and it was attributed to the reduction of thermal damage on the surface of PAN during the stabilization process. The DSC results indicated that the cyclization enthalpy and temperature of F-Ph-CNT/PAN composite were much lower than those for homo PAN. The integration of SWCNTs into polyaniline fibers (PANi) in wet spinning was investigated by Mottaghitalab et al. [21] The addition of CNT in N,N’-Dimethyl propylene urea (DMPU) to PANi was found to limit aggregation of CNTs. By incorporating 2 wt% CNTs to PANi, its yield stress, tensile stress, and Young’s modulus were improved. Chitosan/PANi/SWCNT fibers were fabricated by wet spinning method by Spinks et al. [22] The conductivity of the synthesized material (10−4 S/cm) was 3 to 4 order of magnitude higher than those of the PANi/chitosan composite film. The mechanical properties were improved and the actuation strains (0.3%) were reduced due to the stiffening effect of the nanotubes. Tensile strength of chitosan/PANi/SWNT fibers was 2 fold of pure chitosan, and elongation at break were increased by 60%.

PPy is one of the commonly studied conducting polymers due to its low actuation voltage, high conductivity and biocompatibility [23,24]. Its actuation voltage is lower than 2 V, reach up to 30 MPa active stress and 39% maximum strain [25–27]. It reaches to the largest stroke value among all the conducting polymers for an electrochemical actuation [28]. PPy actuators have been synthesized by a variety of methods. Electrodeposition [29], drop casting [19] and electrospinning [30] are the most common methods. Electrospinning method provides extremely rapid formation of the nanofiber structure, high material elongation rate and a cross-sectional area reduction [31]. Because of its poor solubility, pure PPy cannot be electrospun, therefore PPy is used generally with a co-polymer such as poly ethylene oxide (PEO) [32], PAN [33], polyvinylidene difluoride (PVDF) [34] and poly strene (PS) [35]. In general, PPy based coatings containing nanofibers were investigated to obtain linear actuators. Ketpang et al. [34] synthesized PVDF/CNT electrospun nanofibers, then they coated those nanofibers by vapor-phase polymerization of PPy. Ju et al. [36] synthesized PAN/CNT electrospun nanofibers and coated with PPy by in situ chemical polymerization.

In the present study, the preparation of CNT incorporated PAN/PPy electrospun nanofibers was investigated. As-grown and functionalized CNTs with various amounts were added to PAN/PPy blends. Their morphological and electrochemical properties were characterized. These composite nanofibers are expected to find use in tissue engineering as scaffold for different cell types with their 3-dimensional structure and they can be utilized as actuators through the actuation effect of PPy and CNTs.

## 2. Materials and methods

### 2.1. Materials

To synthesize PAN/PPy nanofibers, PAN (MW = 150000), PPy (conductivity >0.005 S/cm) and solvent N,N-dimethylformamide (DMF) were purchased from Sigma-Aldrich Chemie GmbH (Taufkirchen, Germany). H_2_SO_4_ (95-97%), HNO_3_ (≥65%), HCl (≥32%) and NH_4_OH were also purchased from Sigma-Aldrich Chemie GmbH and applied for CNT functionalization. All these reagents were used without further purification.

### 2.2. CNT growth, purification, functionalization, and characterization

CNTs were produced on Co-Mo/MgO catalyst as reported in our previous study [37]. Before CNT growth, Co-Mo/MgO catalyst was pretreated under 200 sccm H2 flow for 1 h at 850 °C. Then, at CNT growth temperature of 1000 °C, CH_4_ gas as a carbon source was fed to the system to initiate CNT growth and the growth lasted for 40 min. At the end of growth time, CH_4_ flow was terminated and the system was left for cooling under H_2_ flow. Growth was performed at atmospheric pressure.

As-grown CNT weighing 200 mg was purified by mixing it in 30 ml HNO_3_ at 60 °C for 30 min to remove MgO and the other catalyst components. Then, CNTs were washed with distilled water several times until the solution became neutral and dried at 100 °C. For functionalization, purified CNTs were sonicated in a mixture of H_2_SO_4_ and HNO_3_ with volume ratio of 3:1 (30 mL/10 mL) for 2 h, and then kept in this solution for 24 h. Subsequently, 20 mL HCl was added to the solution and it was sonicated again for 2 h. Finally, ammonium hydroxide was added to the solution in a controlled manner to neutralize the solution. After neutralization, CNTs were filtered and washed with deionized water until the pH was 5.5 [38].

As-grown and functionalized CNT samples were characterized by scanning electron microscopy (SEM), energy-dispersive X-ray spectroscopy (EDX), Raman spectroscopy and Fourier transformed infrared spectroscopy (FTIR).

### 2.3. PAN/PPy nanofibers preparation by electrospinning and their characterization

PAN/PPy nanofibers were synthesized by electrospinning method. PPy was taken as a conducting polymer. It is difficult to synthesize pure PPy nanofibers via electrospinning method because of its poor solubility and high 30. To enhance its poor solubility and mechanical properties, PAN was used as a host polymer in the present study.

The electrospinning solution was prepared by dissolving PAN in DMF (8 wt%) until a clear solution was obtained by mechanical stirring. To this solution, PPy amounting 10 and 25 wt% of the total polymer mass was added, and then it was stirred for 3 days at 60 °C. For CNT containing fibers preparation, different contents of as-grown CNT (1, 2, 3 and 4 wt% of the total polymer mass) and functionalized CNTs (1 and 4 wt%) were added into the PAN/PPy solution. They were dispersed in ultrasonic bath for 24 h.

The resultant solution was filled into a 20 mL syringe connected to a high voltage for electrospinning. For all samples, 15 kV voltage was applied and flow rate of solution was kept at 1.5 mL/h. The distance between the syringe and collector was kept as 30 cm and the fibers were collected on an Al foil.

The morphology and diameter of PAN/PPy and PAN/PPy/CNT nanofibers were characterized by SEM (FEI QUANTA 250 FEG) with an acceleration voltage of 5 kV and a secondary-electron detector. For this purpose, the samples of dried nanofibers were fixed onto metallic stubs with double-sided carbon tape. Micro structure and defects determination of CNT embedded nanofibers were determined by TEM. Tensile tests of nanofibers were performed by using a TA.XT plus texture analyzer with a 5 kg load cell and 0.1 mm/s test speed. For tensile tests, sample dimensions were kept as 8 ×60 mm.

Electrochemical impedance spectroscopy (EIS) measurements of the nanofibers were performed by using a three-electrode electrochemical cell: working electrode was graphite electrode covered with our nanofibers during electrospinning process, reference electrode was Ag/AgCl and counter electrode was a Pt wire. The electrolyte solution was Fe[CN6]3-/4 solution.

## 3. Results and discussion

### 3.1. Features of the CNTs produced

**Figure 1:**
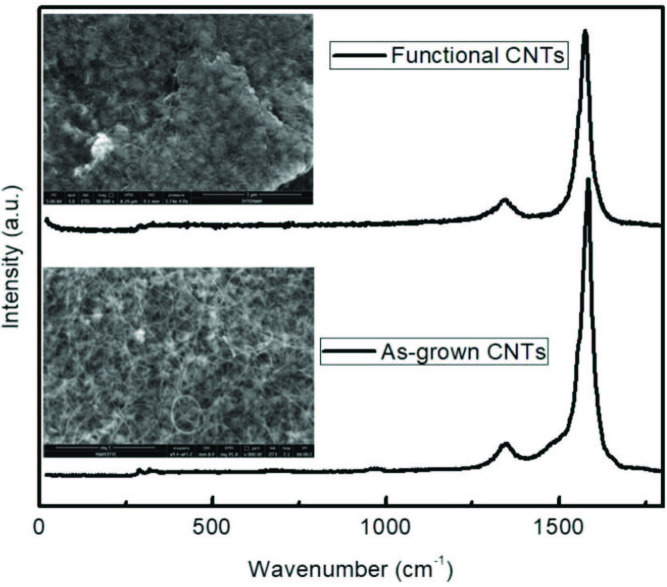
Raman spectra of as-grown and functionalized CNTs.

Raman spectra of the CNT samples given in Figure 1 displays the characteristic peaks of CNTs; defect-derived D-band (about 1300 cm^-1^) and graphitization-derived G-band (1574 cm^-1^). The high intensity of the G band with respect to D band of as-grown CNTs indicated low defect density and high crystallinity. After acid treatment, these characteristic peaks of CNTs were still present, which indicated that acid treatment did not damage the structure of CNTs. However, after functionalization ID /IG ratio was increased. This might be due to breakage of some bonds of CNTs and chemical groups bonded to these groups after H_2_SO_4_/HNO_3_/HCl treatments. These chemical groups could be interpreted as defects on the structure, which lead to increase in D band intensity. SEM images of the as-grown and functionalized CNT samples displayed that as-grown CNTs were straight, nontangled and individual. However, functionalized CNTs were observed to be in the form of CNT groups instead of individual CNTs.

The elemental composition of as-grown and functionalized CNTs are given in Table 1. EDX is an important characterization technique to define the content of oxidized CNTs and elemental composition. EDX results were consistent with Raman results. As-grown CNTs contained a large amount of catalyst components. After functionalization by HNO_3_/H_2_SO_4_ /HCl acids, almost all of the Mg was removed and amount of oxygen increased.

**Table 1:** Elemental composition of as-grown, purified and functionalized CNTs.

Element (wt%)	As-grown CNTs	Functionalized CNTs
C	75.6	80.8
O	9.6	14.0
Mg	11.4	0.1
Co	1.6	2.1
Mo	1.8	3.0

**Figure 2:**
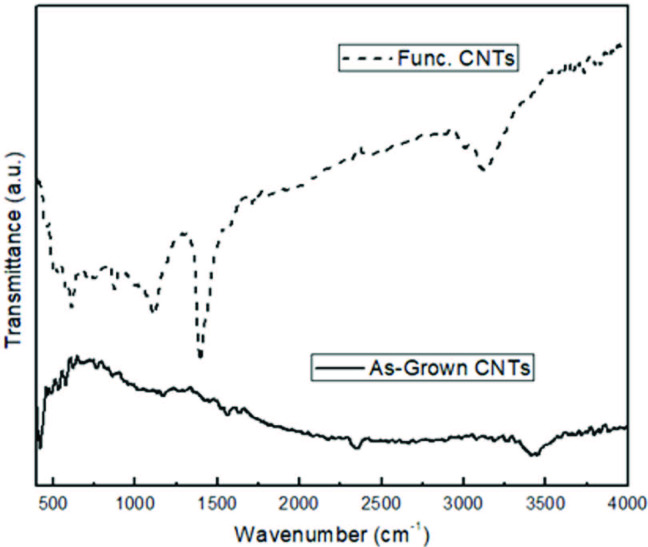
FT-IR spectra of as-grown and functionalized CNTs.

The presence of functional groups on the nanofiber surfaces after acid treatment determined by FT-IR spectroscopy (Figure 2). The high symmetrical structure of CNTs gave very weak infrared signals because charge state between carbon atoms do not show significant difference and this small difference of charge state causes very small induced electric dipole [38]. The peaks between 2800 and 3500 cm^-1^ are characteristic peaks of C-H and O-H bonds after purification and functionalization processes. These peaks may be associated with carboxylic and hydroxylic groups. The peak appearance after functionalization indicated that stretching OH was from hydroxylic group after treated with H_2_SO_4_/HNO_3_/HCl. The functionalized CNTs also gave a peak at about 1475 cm^-1^ which was associated with the C-O stretching. This indicated the presence of carboxylic groups due to surface oxidation. The FT-IR spectra confirmed the presence of functional groups after acid treatment.

### 3.2. PAN/PPy and CNT embedded PAN/PPy nanofibers

PPy is a conducting polymer and it increases electrospinning solution conductivity. Solution conductivity is a significant parameter for morphology of electrospun nanofibers. When a polymer solution with high electrical conductivity is utilized, an electrical force which elongates the polymer jet emerging from Taylor cone is created and it provides formation of uniform fibers with smaller diameter. However, if the solution is very conductive, it will be unstable in electrical field and causes significant bending of the polymer jet at Taylor cone and fiber diameter will become larger [39]. Radius of the polymer jet is inversely proportional with the cube root of the conductivity of the polymer solution [40]. In literature Chronakis et al. studied PPy/PEO electrospun fibers [30]. They indicated that a crytical amount of PEO should be inserted into PPy solution to obtain a fibrous structure. Ji et al. [41] studied PPy with PAN and PAN/PPy bicomponent nanofibers exhibited increasing disordered structure with the increase of PPy concentration and intermolecular interactions were formed between PAN and PPy during nanofiber formation. It was reported ordered and beadless structure of PAN/PPy nanofibers with 10 and 25wt% of PPy contents previously [42]. Increasing PPy content resulted in an increase in solution conductivity and a decrease in solution viscosity, therefore producing nanofibers with smaller diameters [23, 43, 44].

PPy has very poor mechanical properties and exhibits a brittle behavior. Therefore, in this study, PAN was used as a host polymer for PPy to improve its mechanical properties. Tensile tests were performed to measure the mechanical properties of PAN/PPy nanofibers. Table 2 shows the tensile properties of the PAN/PPy nanofibers containing 10 and 25 wt% of PPy. The tensile strain at failure of the nanofibers containing 10 wt% of PPy was much higher than those for 25 wt% of PPy. This was attributed to the brittle nature of PPy. However, the tensile strength of nanofibers were not significantly affected by PPy content.

**Table 2:** Average diameters and tensile properties of the PAN/PPy nanofibers containing 10 and 25 wt% PPy.

PPy amount (wt%)	Average diameter (nm)	Tensile strain (%)	Tensile strength (MPa)	Contact angle (°)	*Viscosity (Pa.s) (×10^-2^)
10	268	23.3	9.3	32.8	10.7
25	153	1.4	9.9	39.4	4.94

*Viscosity values of the solutions containing different amounts of PPy at shear rate of 400 s^-1^.

PPy is a hydrophobic material. However, as a copolymer PAN affected the surface properties and electrospun PAN/PPy nanofibers exhibited a hydrophilic property in water, as observed on contact angle measurements given in Table 2. As increasing PPy amount from 10 to 25%, contact angle value increased about 7o .

The polymer solution viscosity also has a significant influence for uniformity and diameter of obtained fibers. The viscosity values of the solutions measured are given in Table 2. The viscosity of prepared solution containing 10wt% PPy was measured to be about 2 times higher than those of the solution containing 25wt% PPy. Increasing PPy amount in solution decreased the solution viscosity. So, higher solution viscosity resulted with larger PAN/PPy nanofibers in diameter [42] because it is more difficult to overcome surface tension for electrical forces at higher viscosity values. It was observed that a high viscosity solution prevented continuous and ordered fiber formations.

**Figure 3:**
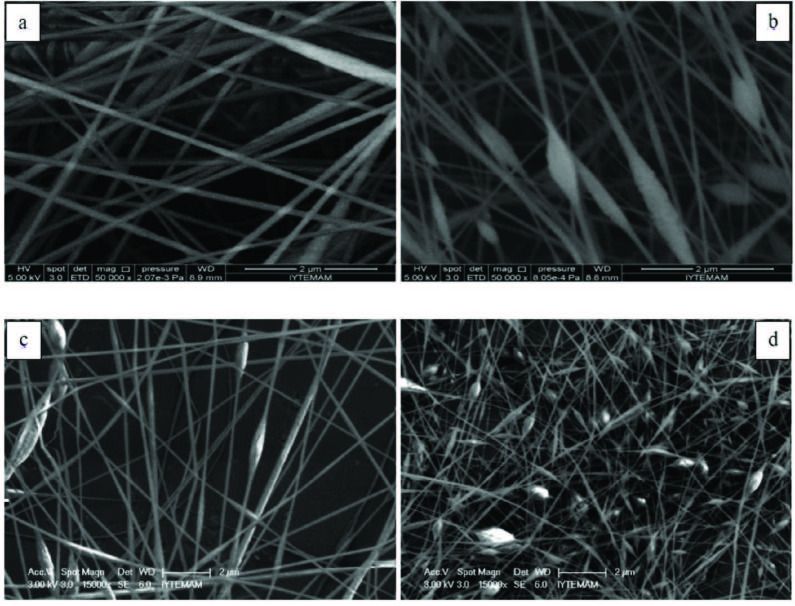
SEM micrographs of the electrospun PAN/PPy nanofibers containing 10 wt% PPy with (a) 1, (b) 2, (c) 3, and (d) 4 wt% of as-grown CNT additions.

**Figure 4:**
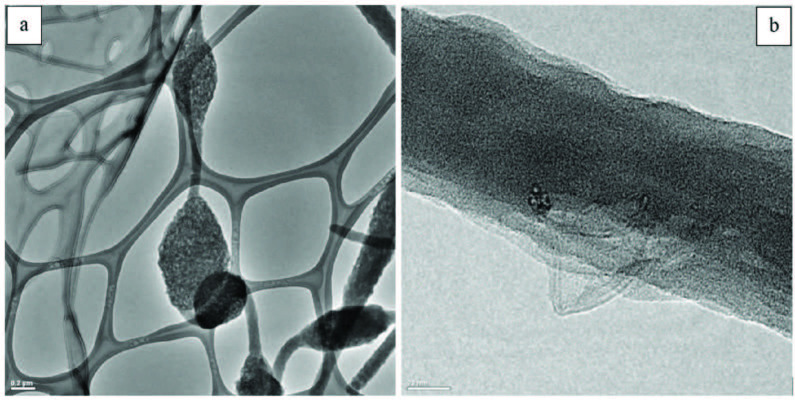
TEM images of (a) CNT agglomeration on electrospun nanofibers, (b) surface irregularities formed due to CNTs not fully embedded within the polymer matrix. The nanofiber sample is prepared from10 wt% PPy/PAN blend and 4 wt% CNT.

Two different amounts of CNTs (1 and 5 wt%) were added into the PAN/PPy solutions containing 25% PPy and then the fibers were electrospun from the prepared solutions. Nanofiber formation was affected by the addition of CNTs in two different ways. The first effect of CNT incorporation was increament in the solution conductivity, the second effect is CNT addition broke the solution homogenity. As a result, some nanofibers were achieved with 1 wt% of CNTs addition, although they contained many beads and irregularities. On the other hand, almost no fiber formation was observed for 5 wt% CNT content. Only discrete beads were formed in this case which might be due to high solution viscosity and agglomeration of CNTs. To obtain better nanofibers with CNTs, PPy content was decreased to 10 wt% and different amount of as-grown CNTs were added (1, 2, 3 and 4 wt%). SEM micrographs of these samples are shown in Figure 3. For 1 wt% CNT content, obtained electrospun nanofibers were smooth and almost no bead formation was observed. However, increasing CNT content terminated continuous spinning and as a result, number of beads increased dramatically. Besides, with increasing CNT content average dimeter of nanofibers were obtained as 95.27, 85.33 nm, 81.77 nm and 69.94 nm for the CNT amount of 1, 2, 3, and 4 wt%, respectively. It was indicated that CNT incorporation decreased the average dimeter of nanofibers. When the bead diameter was analysed, for 2 wt% CNT content the average bead dimeter was 345.76, for 4 wt% CNT amount, the average bead diameter desreased to 217.74 nm. As a result, with the increasing amount of CNT, number of beads increased, however, fiber and bead diameter decreased. In literature Kaur et al studied CNT incorporated PAN nanofibers and they also reported nanofiber diameter decrease with CNT addition [45]. It was observed that nanofibers with 4 wt% CNT sample contained a high number of beads and disordered features on their surfaces. TEM images of this sample showed that some of the CNTs were not fully embedded into the nanofibers and they agglomerated either forming beads or simply stuck to the surface of the fibers (Figure 4).

**Figure 5:**
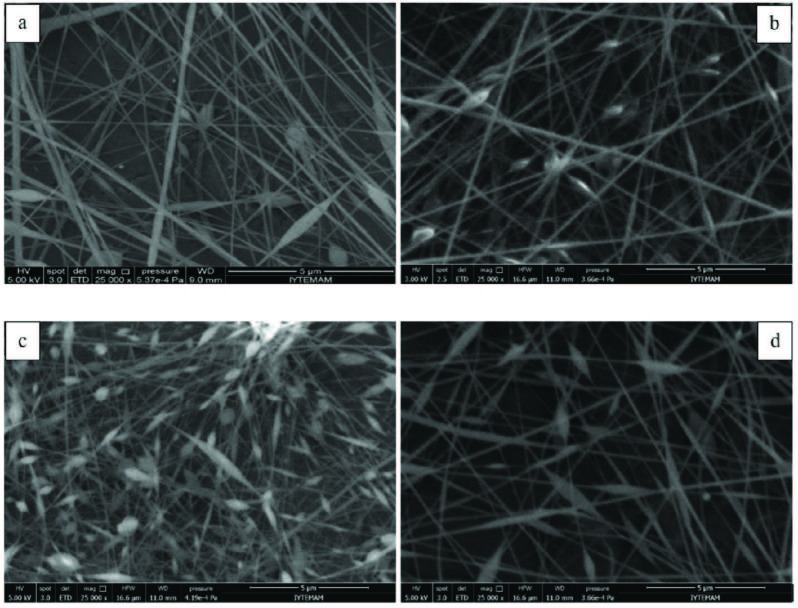
SEM micrographs of PAN/PPy/CNT electrospun nanofibers containing 10 wt% PPy and (a) 1wt% as-grown CNT embedded, (b) 1wt% functional CNT embedded, (c) 4wt% as-grown CNT embedded, (d) 4wt% functional CNT embedded.

To further improve nanofiber formation, functional CNTs (1 and 4 wt%) were added to 10 wt% PAN/PPy mixture. The SEM micrographs of the PAN/PPy/CNT nanofibers prepared with functional CNTs are given in Figure 5. For 1 wt% of functional CNTs, the amount of nanofibers produced were higher and the numbers of beads were lower, as compared to the electrospun nanofibers prepared with as-grown CNTs. Similarly, a better nanofiber formation was observed for 4 wt% functional CNTs compared to as grown CNTs. When the average diameter of nanofibers containing functional CNTs were examined; for 1 wt% CNT amount, average diameter was 92.16 nm and very little increase was observed compared to nanofibers containing 1 wt% as-grown CNTs. For 4 wt% CNT amount, the average diameter was 73.84 nm. Here, a little decrease was observed in average dimater compared to the sample containing as-grown CNTs. Functionalization of CNTs was not significantly influenced fiber diameter. It was revealed in this study that functionalization of CNTs improved electrospun nanofiber formation significantly.

**Figure 6:**
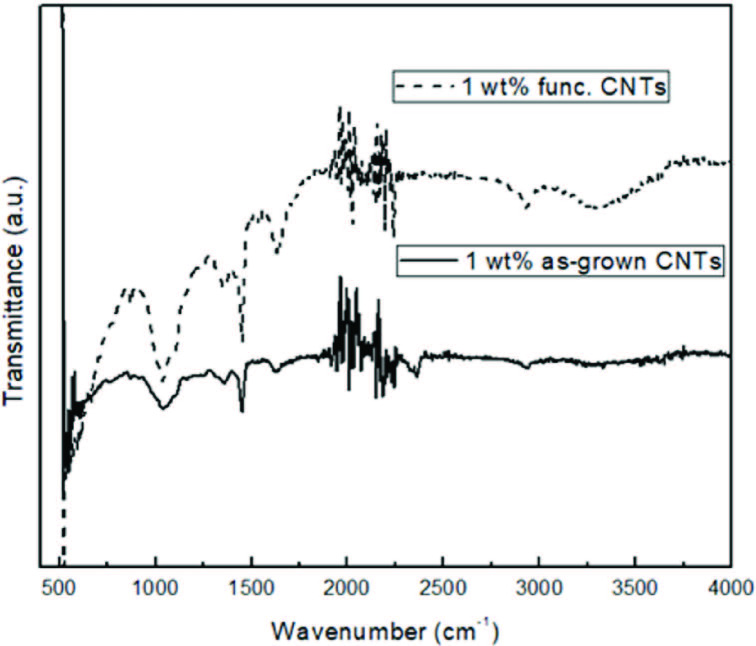
FT-IR spectra of PAN/PPy/CNT nanofibers containing 1 wt% as-grown and functionalized CNTs.

Figure 6 shows the FT-IR spectra of CNT embedded PAN/PPy nanofibers. Peaks observed at 1454 cm^-1^ and 2354 cm^-1^ are the characteristic peaks of PAN and the peaks at 1270 cm^-1^ and 1654 cm^-1^ are induced by C-N and C=N stretching, respectively. The peak at 1454 cm^-1^ and 1068 cm^-1^ corresponded to the C=C and C-H. This data confirmed the PPy existence in PAN. Addition of as-grown CNTs did not change the FT-IR spectrum of PAN/PPy nanofibers due to the symmetrical structure of as-grown CNTs. However, sample including functionalized CNTs showed a broad peak at about 3400 cm^-1^ which was a characteristic of the O-H stretching of hydroxylic group. Peaks between 2800 and 3500 cm^-1^ were characteristic peaks of C-H and O-H bonds which could be related to carboxylic and hydroxylic groups.

**Figure 7:**
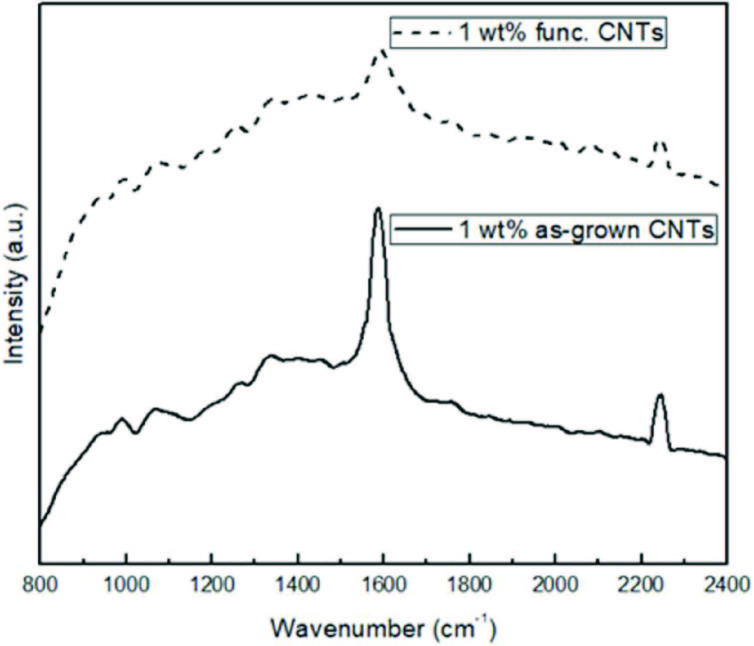
Raman spectra of PAN/PPy/CNT nanofibers containing 1 wt% as-grown and functionalized CNTs.

Raman spectra of the PAN/PPy/CNT electrospun nanofibers containing 1 wt% CNT is displayed in Figure 7. D and G peak were observed at about 1300 cm^-1^ and 1590 cm^-1^, respectively. Nitrile group (-CN) peak was observed at 2240 cm−1 . ID/IG ratio was significantly low if as-grown CNTs were utilized due to highly graphitized CNTs. ID/IG value increased with the addition of functional CNTs. Graphitization decreased with functionalized CNT addition because of broken bonds after acid treatment.

**Figure 8:**
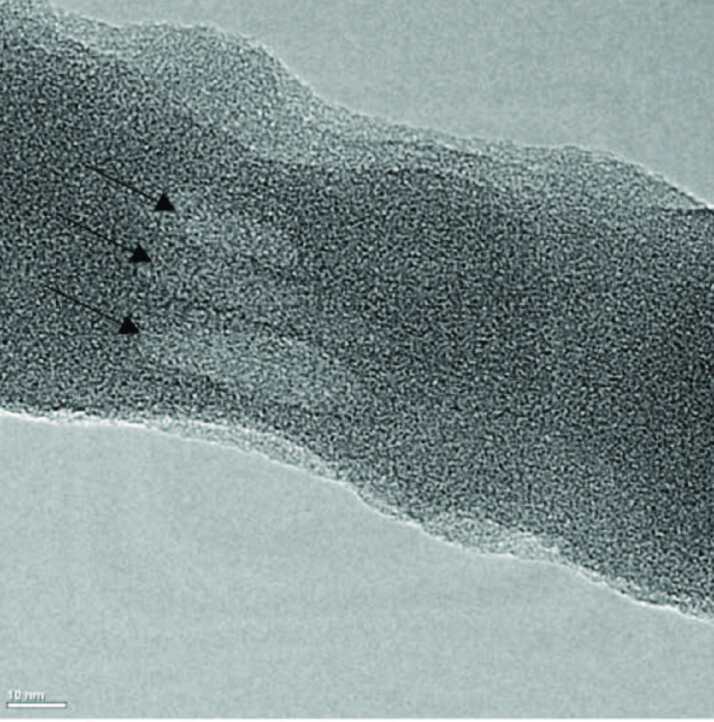
TEM images of PAN/PPy nanofibers containing 10 wt% PPy and 1 wt% functional CNTs.

The concentric tubular structure of functional CNTs could be clearly observed in TEM images of the nanofibers (Figure 8). They were dispersed uniformly and aligned along the direction of nanofibers.

**Figure 9:**
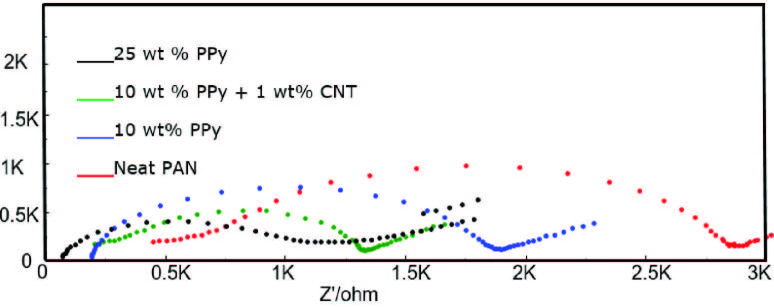
Electrochemical impedance spectra of the PAN/PPy nanofibers for 10 wt% PPy+1 wt% CNT, 25 wt% PPy and 10 wt% PPy.

EIS data give information about the nature of electrochemical process occurring at the electrode/electrolyte interface. Analysis of the interficial properties such as resistance and capacitance of the sample are combined by EIS by applying a very small amplitude sinusoidal voltage. During EIS measurements electrospun nanofibers displayed electroactivity, they remained stable and no deformation or decomposition was observed in their structures. The Nyquist graphs of PAN/PPy nanofibers were given in the frequency range of 0.01 Hz to 100 kHz (Figure 9). Nyquist plots of PAN/PPy nanofibers exhibited semicircles. The diameter of the semicircle represents the charge-transfer resistance (Rct) of PAN/PPy nanofibers .()[46]. Rct of nanofibers decreased with inceasing PPy amount which depends on the change in charge of the surface of PAN/PPy nanofibers. With 25 wt% PPy content Rct decreased significantly. CNT addition in PAN/PPy nanofibers also decreased Rct value of nanofibers as given in Table 3. The impedance value of nanofibers calculated from Nyquist plots decreased with increasing PPy amounts as given in Table 3. While PAN nanofibers showed a 2603 ohm Rct value, Rct values were 1688 and 1066 ohm for PPy ratios of 10 and 25 respectively. It means the highest PPy content showed the highest electrical conductivity. CNT addition also decreased the Rct value from 1688 to 1481 ohm. As a result, increasing PPy and CNT addition decreased Rct and improved electrical properties of nanofibers. PPy as a conducting polymer and CNT as a conducting nanomaterial enhanced the conductivity of nanofibers, therefore the decrease in Rct values was observed with the addition of these materials as expected.

**Table 3:** The impedance values of PAN/PPy nanofibers for different amounts of PPy.

Sample	PAN	PP1	PP3	PPC3
R_ct_ (ohm)	2603	1688	1066	1481

## 4. Conclusion

PAN/PPy and CNT added PAN/PPy nanofibers were prepared by electrospinning method. The diameter of the nanofiber decreased by increasing PPy content in PAN/PPy. The nanofibers with low PPy amount were found to be more ductile. The nanofibers with beads and agglomerated CNTs were observed when as-grown CNT was added to PAN/PPy. With increasing CNT content, extensive bead formation and disordered sites were observed on nanofibers. The formation of electrospun nanofibers became more difficult at high PPy and CNT contents. This was probably due to agglomeration of CNTs, high solution viscosity and conductivity. Functionalization of CNTs enhanced the dispersion of CNTs within the nanofibers, and hence CNT alignment along the direction of nanofibers under high electrical field. In order to obtain more homogen nanofibers with higher tensile strain and higher hydrophilicty, PPy should be utilized low contents in PAN solution in DMF and CNT incorporation also should be kept at low contents and functionalized CNTs shold be used. The EIS results indicated that CNT addition and increasing PPy amount in nanofibers decreased the resistance value and enhanced electrical properties of nanofibers.
